# An impedance pneumography signal quality index: Design, assessment and application to respiratory rate monitoring

**DOI:** 10.1016/j.bspc.2020.102339

**Published:** 2021-03-01

**Authors:** Peter H. Charlton, Timothy Bonnici, Lionel Tarassenko, David A. Clifton, Richard Beale, Peter J. Watkinson, Jordi Alastruey

**Affiliations:** a Department of Biomedical Engineering, School of Biomedical Engineering and Imaging Sciences, King’s College London, King’s Health Partners, London SE1 7EH, UK; b Institute of Biomedical Engineering, Department of Engineering Science, University of Oxford, Oxford OX3 7DQ, UK; c Primary Care Unit, Department of Public Health and Primary Care, University of Cambridge, Strangeways Research Laboratory, Worts’ Causeway, Cambridge CB1 8RN, UK; d Department of Asthma, Allergy and Lung Biology, King’s College London, King’s Health Partners, London SE1 7EH, UK; e Nuffield Department of Medicine, University of Oxford, Oxford OX3 9DU, UK; f Kadoorie Centre for Critical Care Research and Education, Oxford University Hospitals NHS Foundation Trust, Oxford OX3 9DU, UK

**Keywords:** Thoracic impedance, Signal processing, Breathing rate, Patient monitoring

## Abstract

Impedance pneumography (ImP) is widely used for respiratory rate (RR) monitoring. However, ImP-derived RRs can be imprecise. The aim of this study was to develop a signal quality index (SQI) for the ImP signal, and couple it with a RR algorithm, to improve RR monitoring. An SQI was designed which identifies candidate breaths and assesses signal quality using: the variation in detected breath durations, how well peaks and troughs are defined, and the similarity of breath morphologies. The SQI categorises 32 s signal segments as either high or low quality. Its performance was evaluated using two critical care datasets. RRs were estimated from high-quality segments using a RR algorithm, and compared with reference RRs derived from manual annotations. The SQI had a sensitivity of 77.7 %, and specificity of 82.3 %. RRs estimated from segments classified as high quality were accurate and precise, with mean absolute errors of 0.21 and 0.40 breaths per minute (bpm) on the two datasets. Clinical monitor RRs were significantly less precise. The SQI classified 34.9 % of real-world data as high quality. In conclusion, the proposed SQI accurately identifies high-quality segments, and RRs estimated from those segments are precise enough for clinical decision making. This SQI may improve RR monitoring in critical care. Further work should assess it with wearable sensor data.

## 1 Introduction

Respiratory rate is a key marker of the progression and severity of acute illness [1]. Consequently, respiratory rate (RR) is routinely monitored in acutely- and critically-ill hospitalised patients [2]. It is often estimated from the thoracic electrical impedance pneumography (ImP) signal, from which individual breaths can be identified and RR can be estimated [3,4]. However, the ImP signal is susceptible to motion artifact [5], and RRs estimated from ImP signals have been found to be imprecise and inaccurate in several studies [6–9]. Erroneous RRs could adversely impact clinical decision making with true clinical deteriorations being missed, or false alerts of deteriorations being raised [10]. Therefore, methods for improving the performance of ImP-based RR monitoring could improve patient safety and reduce resource utilisation.

A common approach to improve the estimation of parameters from physiological signals is to use a signal quality index (SQI) to identify segments of high quality signal, from which parameters can be more reliably estimated [11]. SQIs have been used to improve: (i) heart rate estimation from the electrocardiogram signal [11]; (ii) pulse rate estimation from the photoplethysmogram signal [12]; and (iii) cardiac output estimation from the arterial blood pressure signal [13]. However, little research has been conducted on the development of a SQI for RR estimation from the ImP signal. Previous studies of ImP signal processing techniques have focused on: motion artifact detection [14] and removal [15,16]; suppressing cardiac content [17]; decomposition into cardiac and respiratory components [18]; and calibration for respiratory parameter estimation [19]. Furthermore, a technique for signal quality assessment has been developed [10,20], which when coupled with a RR algorithm was found to have a bias and limits of agreement of 1.7 ± 5.8 bpm in the challenging environment of patient transport [20]. In addition, a technique termed the agreement SQI has been developed to determine whether a segment of ImP signal is deemed to be of sufficiently high quality to estimate RR from it accurately [21,22]. Whilst this technique has been applied to data from healthy volunteers and hospitalised patients, its performance has not been assessed, and was found to be sub-optimal in this work. Therefore, there is scope for developing a novel SQI to improve the performance of ImP-derived RRs in the hospital setting.

The choice of algorithm to estimate RR from physiological signals also affects the performance of RR monitoring [23]. Different RR algorithms have been shown to have different performances when applied to the electrocardiogram and photoplethysmogram signals [24], and the ImP signal [25]. Indeed, a comparison of eight different RR algorithms applied to ImP signals acquired at rest found a fourfold increase in mean absolute error between the best and worst performing algorithms (which were either based on detecting individual breaths in the time domain, or identifying the frequency corresponding to the maximum spectral power in the frequency domain) [25]. Therefore, it is important to ensure that a high-performance RR algorithm is used for ImP-based RR monitoring.

The aim of this study was to develop a novel SQI, and couple it with a high performance RR algorithm, to improve the performance of ImPbased RR monitoring in the hospital inpatient setting. The novel SQI identifies candidate breaths and assesses signal quality using: the variation in detected breath durations, how well peaks and troughs are defined, and the similarity of breath morphologies. It categorises signal segments as either high or low quality. The first objective of this study was to assess the discriminatory performance of the novel SQI for distinguishing between high and low quality ImP signal segments. The second objective was to compare the accuracy and precision of RRs obtained from high quality segments identified by the novel SQI when using: (a) RRs reported by a clinical monitor; and (b) RRs estimated using a high performance RR algorithm. The third objective was to assess the frequency at which RRs are reported when using the novel SQI in a real-world setting. The novel SQI was compared with the agreement SQI, and the generalisability of the findings was assessed using a second independent dataset. In Section 2 the datasets used in this study are described, as well as the signal processing methods, and the analysis methods. The results are presented in Section 3. The potential implications of this work for clinical practice are discussed in Section 4. This study builds on the work presented in [23,26], in which the algorithm design was presented, but its performance was not assessed on separate datasets.

## 2 Materials and methods

### 2.1 Datasets

Two datasets were used in this study: the *RRest-vent* dataset and the *MIMIC* dataset. Both datasets contain ImP signals, which were split into adjacent 32 s segments for analysis. A duration of 32 s was chosen as a compromise between a longer duration (which in other applications has been found to improve RR estimation) and a shorter duration (which could allow shorter high quality segments to be identified between transient artifacts and allow changes in RR to be tracked more accurately) [27].

The *RRest-vent* dataset was used to: (i) develop the SQI; (ii) assess its discriminatory performance; (iii) compare the accuracy and precision of RRs reported by a clinical monitor, and estimated by a RR algorithm; and (iv) assess the impact of false positives (segments falsely identified as high quality by the novel SQI) on RR estimates. This dataset, described in [23], contains data from 59 hospital patients during their recovery from major cardiac surgery at St Thomas’ Hospital (London, UK). The following data were recorded from the clinical monitors (IntelliVue MP70, Philips Medical Systems, Andover, MA, USA) used as part of routine post-operative care: ImP signals at a sampling rate of 125 Hz, and the RRs estimated by the monitor from its ImP signals at 1 Hz. Data were acquired throughout patients’ critical care stay using BedMaster data acquisition software (v.4.1.12, Excel Medical Electronics, Jupiter, FL, USA). Data were extracted at three time points during each patient’ s stay: whilst mechanically ventilated on the intensive care unit (ICU, in the hour immediately prior to disconnection from the ventilator); unassisted on ICU (within the hour after disconnection); and, shortly before discharge from critical care to the ambulatory ward (within the eight hour period leading up to discharge). A total of 29.5 h of data were extracted, consisting of 10 min of data (18 segments) at each time point for each patient. This dataset is a subset of the LISTEN dataset, which was collected in accordance with the Declaration of Helsinki as part of National Clinical Trial no. 01549717, and approved by the Bloomsbury Research Ethics Committee (reference 12/LO/0526) [28]. Patients provided informed consent to participate in the study. The 59 patients studied in this work were selected from the wider LISTEN dataset using the following criteria: (i) patients had to follow a typical recovery trajectory (moving from intensive care to a high-dependency unit, to an ambulatory ward) and be in sinus rhythm during the recording periods; and (ii) patients were excluded if they were paced, or if a full set of physiological signals could not be recorded from the monitor.

The *RRest-vent* dataset was split into training and testing subsets. The training subset, consisting of data from 34 subjects, was used to design the novel SQI. The testing subset, consisting of data from the remaining 25 subjects, was used for analysis. Each of the 1,350 segments in the testing subset was manually labelled as being either high or low quality, where a “high quality” label was only given if the single expert annotator was confident that all the breaths in that segment could be accurately identified. In addition, individual breaths were annotated in those segments deemed to be of high quality in any of: the manual annotations, the novel SQI, or the agreement SQI. These were used to calculate reference RRs (calculated as the mean breath duration) to compare with those estimated from the ImP signal.

The *MIMIC* dataset was used to: (i) compare the accuracy and precision of RRs reported by a clinical monitor, and estimated using a RR algorithm; and (ii) assess the frequency at which RRs are reported when using the novel SQI in a real-world setting. For this study, a single hour of data from each of 100 adult critical care patients at the Beth Israel Deaconess Medical Center (Boston, MA, USA) was extracted from the *MIMIC-III Waveform Database Matched Subset* [29,30]. The resulting dataset was termed the *RRest-mimic* dataset. Patients were continuously monitored using the same clinical monitors as in the *RRest-vent* dataset [31]. Similarly, ImP signals sampled at 125 Hz and RRs estimated from the ImP signals at 1 Hz, were extracted from the routine monitoring data. Individual breaths were manually annotated in a sample of the segments deemed to be of high quality by the novel SQI: breaths were annotated in five segments for each of the 87 patients with at least five high-quality segments; annotations were made for between one and five segments for 8 patients; and no high quality segments were available to annotate in 5 patients. A total of 452 segments were annotated. The *MIMIC-III Waveform Database Matched Subset* is freely available. Details of how to access the data used in this study (the *RRest-mimic* dataset), and the Matlab ® code used to download and extract the data, are provided in the Data Access Statement.

### 2.2 The novel signal quality index algorithm

**Fig. 1:**
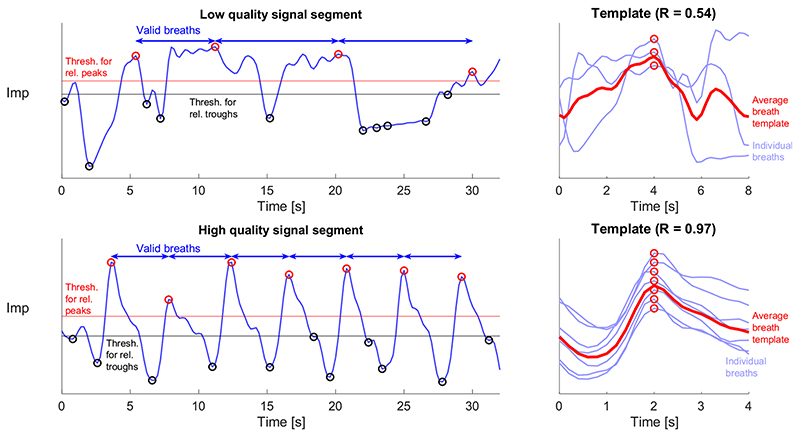
Impedance (ImP) signal quality assessment: A novel SQI algorithm was designed to assess the quality of ImP signal segments. On the left, two ImP segments are shown. Hollow red and black dots indicate relevant peaks and troughs respectively, which were used to identify valid breaths indicated by arrows (as described in Section 2.2). Relevant peaks and troughs were identified using the thresholds shown, with only one relevant peak permitted between consecutive relevant troughs. On the right are the corresponding average breath templates (red, aligned by each relevant peaks) and the individual breaths (blue) from which they were calculated. The upper segment is of low quality, as indicated by a low mean correlation coefficient (R) between the individual breaths and average breath template of 0.54. The lower segment is of high quality, as indicated by a high R of 0.97.

**Fig. 2:**
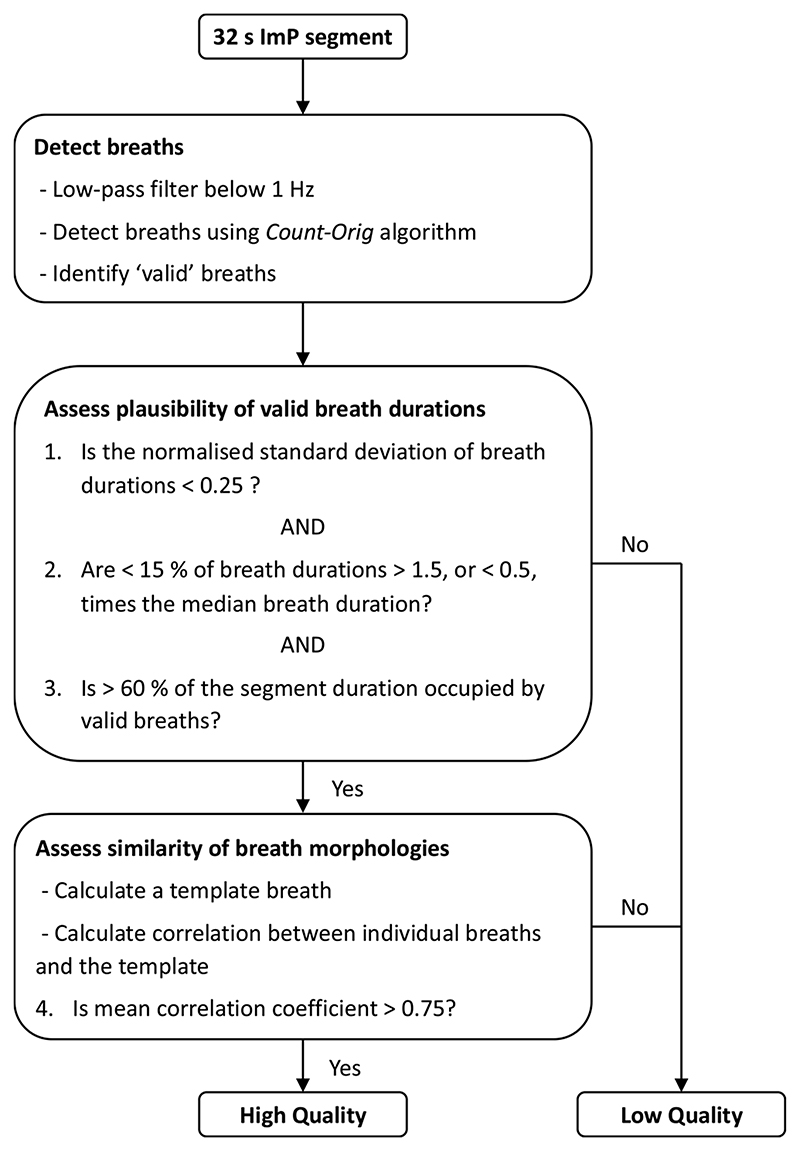
A flowchart of the signal quality index (SQI) algorithm.

The novel SQI was developed by adapting the approach proposed by Orphanidou et al. for cardiac signals in [11]. It takes a 32 s segment of ImP signal as an input, and outputs the signal quality (either high or low), and a RR estimate for high-quality segments. The novel SQI consists of three stages. Firstly, individual breaths are detected in the signal. Secondly, breath durations are assessed for physiological plausibility. Any signal segments with implausible cycle timings were deemed to be of low quality. Thirdly, template matching is used to assess the similarity of breath morphologies as shown in Fig. 1. If the correlation between the average breath’s morphology, and each individual breath’s morphology, is high enough then the signal segment is deemed to be of high quality. The three stages of the SQI are illustrated in Fig. 2, and are now described in detail.

The first stage, detection of breaths, was performed as follows. The ImP signal was low-pass filtered to remove frequency content above 1 Hz (-3 dB cutoff at 1.0 Hz, *i.e*. 60 breaths per minute, bpm) using a Tukey window to avoid edge effects (tapered for 2 s at either end), and downsampled to 5 Hz. Each 32 s segment was normalised to have a mean of 0 and standard deviation of 1; inverted; and any linear trend was removed. Individual breaths were then identified in the ImP signal using a modified version of the *Count-orig* method proposed in [32], containing the following steps:

1. Peaks and troughs were detected as local extrema *(i.e*. points of greater amplitude for peaks, or lower amplitude for troughs, than the two neighbouring points).
2. Relevant peaks were identified as those above 0.2 times the 75th percentile of all peak amplitudes (shown as hollow red dots in the left-hand panels of Fig. 1).
3. Relevant troughs were identified as those below 0.2 times the 25th percentile of all trough amplitudes (shown as hollow black dots in the left-hand panels of Fig. 1).
4. The relevant peaks between each pair of consecutive relevant troughs were identified. If there was more than one relevant peak then only the relevant peak with the greatest amplitude was retained.
5. Valid breaths were identified as the time spanning consecutive relevant peaks with at least one relevant trough between them (indicated by arrows in the left-hand panels of Fig. 1). The duration of each valid breath was calculated as the time between the relevant peaks marking the start and end of that valid breath.

The second stage, assessment of the physiological plausibility of valid breath durations, was performed as follows. Three criteria were used: (i) the normalised standard deviation of breath durations had to be < 0.25 to permit only moderate variation in the durations of detected breaths; (ii) the proportion of breath durations > 1.5, or < 0.5, times the median breath duration had to be < 15 % to prevent errors due to outlying breath durations; (iii) > 60 % of the segment duration had to be occupied by valid breaths. Any segment which did not satisfy these three criteria was deemed to be of low quality.

The third stage, assessment of the similarity of breath morphologies, was performed as follows. First, the mean breath interval was calculated as the mean interval between consecutive relevant peaks. Second, individual breaths were extracted as signal segments of duration equal to the mean breath interval centred on each relevant peak (any individual breaths which extended beyond the start or end of the 32 s segment were discarded), and each normalised by their Euclidean norm. Individual breaths are shown in blue in the right-hand panels of Fig. 1. Third, an average breath morphology template was calculated as the mean of all the individual breaths centred on their relevant peaks (shown as hollow red dots in the right-hand panels of Fig. 1). The similarity of breath morphologies was quantified using the mean correlation coefficient between individual breaths and the average breath template. The correlation coefficient had to be > 0.75 for the signal segment to be considered to be high quality.

The criteria and thresholds used in the SQI were manually chosen from a range of possible values to optimise performance on the *RRest-vent* training subset. This approach resulted in similar performance to automatic determination of thresholds using a linear logistic regression model.

### 2.3 The agreement signal quality index algorithm

The previously proposed agreement SQI was used as a comparator in this study, since it was also originally designed to determine whether RR can be accurately estimated from a segment of ImP signal in the hospital setting [21,22]. The agreement SQI consists of estimating RR using independent time- and frequency-domain techniques, calculating the difference between the two resulting RRs, and concluding that the signal segment is of high quality if and only if the difference is < 2 bpm. In this study the two RRs were estimated by: (i) using the time-domain *Count-orig* method described in Section 2.2; and (ii) calculating the power spectrum of the signal using the Welch Periodogram (analysing 32 s windows with overlapping sections of duration 12.8 s, and 50 % over-lap), and estimating the RR as the frequency corresponding to the maximum power spectral density. These methods were chosen because of their high performance in [23]. Further details on these two methods are provided in [24].

### 2.4 Assessing the discriminatory performance of the novel SQI

The discriminatory performance of the novel SQI for distinguishing between high and low quality signal segments was assessed by comparing its labels of signal quality to the manual annotations on the *RRest-vent* testing subset. Discriminatory performance was quantified using sensitivity and specificity, defined as:

$$\mathrm{sensitivity}=\frac{TP}{P},$$

where *TP* is the number of true positives *(i.e*. segments which were correctly deemed to be of high quality by the SQI), *P* is the number of positives *(i.e*. segments annotated as high quality); and

$$\mathrm{specificity}=\frac{TN}{N},$$

where *TN* is the number of true negatives *(i.e*. segments which were correctly deemed to be of low quality by the SQI), *N* is the number of negatives (*i.e*. segments annotated as low quality). 95 % confidence intervals (CIs) were calculated using bootstrapping with 1000 bootstrap replicas.

The performance of the novel SQI was compared to that of the previously proposed agreement SQI using the two-sided asymptotic McNemar test at the 5% significance level [33]. The statistical measures of discriminatory performance reported for the novel SQI were also reported for the agreement SQI.

### 2.5 Comparing RRs obtained from a clinical monitor and using a RR algorithm

The *RRest-vent* testing subset and the *RRest-mimic* datasets were used to compare the accuracy and precision of RRs obtained from high quality segments when using: (a) RRs reported by a clinical monitor, and (b) RRs estimated using a high-performance RR algorithm. To do so, RRs were obtained from those reported by the clinical monitor by calculating the median of the RRs outputted by the monitor during each segment. The RR algorithm used to estimate RRs from ImP signals was a modified version of the *Count-Orig* algorithm [32], since it has performed well in several previous studies [23–25]. This consisted of identifying valid breaths (as performed in the first stage of the novel SQI, described in Section 2.2), and calculating the RR of each segment as the mean duration of valid breaths in that segment. The reference RR for each segment was calculated as the mean breath duration derived from the manually annotated breaths.

A further subgroup analysis of the *RRest-vent* testing subset was conducted to assess the impact of false positives *(i.e*. low quality segments falsely identified by the SQI as high quality) on RR estimates. The accuracy and precision of RRs estimated using the RR algorithm were calculated for two subgroups of segments: those which were correctly identified as high quality by the novel SQI, and those which were incorrectly identified as high quality (as detemined through manual annotation).

The following statistical methods were used to assess the accuracy and precision of RRs. The agreement between each method’s RRs and the reference RRs was quantified using the Limits of Agreement technique [34]. The accuracy of RRs was quantified using the bias *(i.e*. mean error, corrected for repeated measurements within subjects),

$$\mathrm{bias}=\frac{\sum_{i=1}^{n}\left(\sum_{j=1}^{m_{i}}\left[{\mathrm{RRref}}_{ij}-{\mathrm{RRest}}_{ij}\right]\right)}{\sum_{i=1}^{n}m_{i}}$$

which was calculated as the mean difference between the estimated RRs, *RR_est_*, and the reference RRs, *RR_ref_*, across the *i* = 1, *n* subjects, each of which had *mi* pairs of estimated and reference RRs. The precision of RRs was assessed by calculating the limits of agreement *(i.e*. the expected range of 95 % of errors around the systematic bias), ± 1.96*s*, denoted as *2SD*, where *s* is the standard deviation of the errors. Any segments in which the estimated RR was zero were excluded from the analysis. The method described in [35] was used to account for repeated measurements per subject. *s* was calculated from the total variance: the sum of the within subjects variance, $\sigma^{2}{}_{w}$, and between subjects variance, $\sigma^{2}{}_{b}$, which were estimated using one-way analysis of variance:

$$\begin{array}{l} {\sigma^{2}}_{w}=MS_{\mathrm{residual}}\\ {\sigma^{2}}_{b}=\frac{MS_{\mathrm{subject}}-MS_{\mathrm{residual}}}{\left(\frac{{\left(\sum m_{i}\right)}^{2}-\sum {m_{i}}^{2}}{\left(n-1\right)\sum m_{i}}\right)} \end{array}$$

where *MS_residual_* is the mean square error, and *MS_subject_* is the difference between the mean squares for subjects, and the sums are from *i* = 1, …, *n*.

Two further statistics were used to assess the utility of RRs. The coverage probability, *CP*_2_, is the proportion of high quality segments for which highly useful RRs are returned, defined as being within 2 bpm of the reference RR. The mean absolute error (MAE) was also reported.

**Fig. 3:**
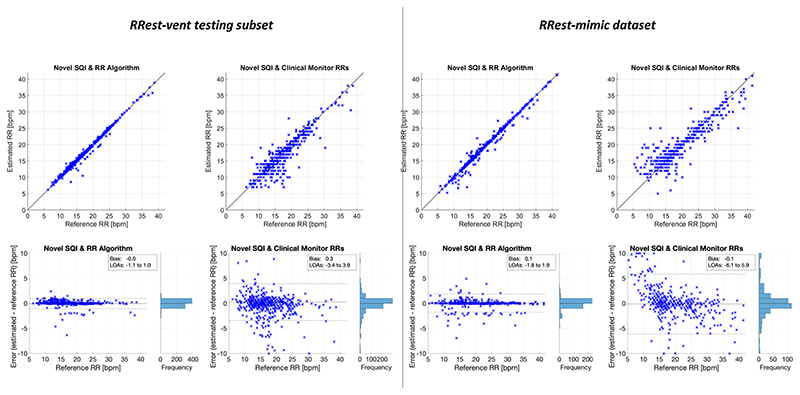
The performance of RRs estimated from segments deemed to be of high quality by the Novel SQI in each dataset. Results are shown for each dataset, and when using the RR algorithm or clinical monitor RRs. Upper plots show the estimated RRs plotted against the reference RRs. Lower plots show the errors against the reference RRs.

Scatter plots of estimated and reference RRs, and Bland-Altman plots of RR errors, were provided (in Fig. 3). Errors of > 10 bpm were truncated to 10 bpm on Bland-Altman plots.

### 2.6 Assessing the frequency at which RRs are reported when using the novel SQI

The frequency at which RRs are reported when using the novel SQI in a real-world setting was assessed using the real-world *RRest-mimic* dataset. Firstly, the proportion of segments which were deemed to be high quality by the novel SQI was assessed, both for the entire dataset and for individual subjects (reported as the median and inter-quartile range). Secondly, the durations of gaps between consecutive high-quality segments were assessed.

**Fig. 4:**
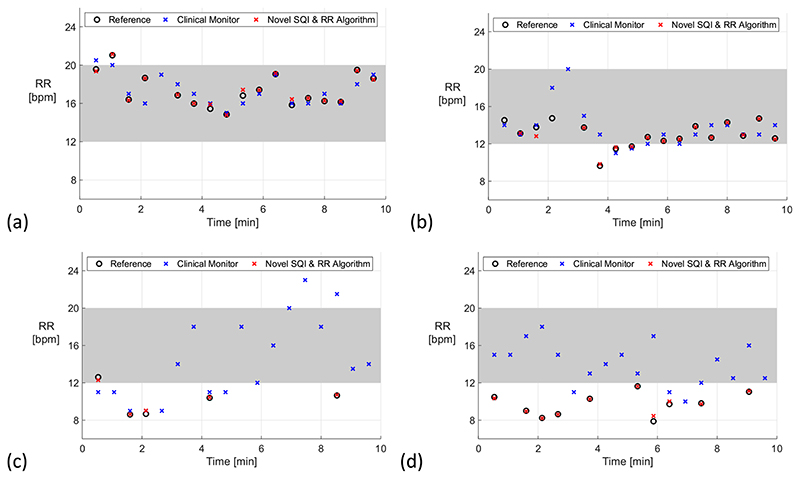
Case studies demonstrating the utility of the novel SQI combined with a RR algorithm (grey shading indicates normal RRs) (a) both the clinical monitor and the novel approach track changes in RR precisely; (b) the clinical monitor outputs a high RR in a period of low signal quality (at 3 min., as indicated by the absence of a reference RR), which could result in a false alert; (c) between 3 and 10 min. the clinical monitor outputs normal RRs in a period of predominantly low signal quality, which may result in an alert being falsely suppressed; (d) the clinical monitor incorrectly outputs mostly normal RRs when the true RRs are low, despite the signal quality being high, which may also result in an alert being falsely suppressed. Data obtained from the RRest-vent dataset.

Case studies were provided to illustrate clinical scenarios in which the novel SQI combined with a RR algorithm may confer clinical benefit over current clinical monitoring (the case studies are provided in Fig. 4).

## 3 Results

### 3.1 The discriminatory performance of the novel SQI

The novel SQI had a sensitivity and specificity (95 % CIs) of 77.7 (74.9−80.4) and 82.3 (79.0−85.2) % respectively on the *RRest-vent* testing subset. The novel SQI classified 79.6 % of the segments correctly, and only 7.3 % of segments were misclassified as high quality when the reference annotation was low quality. The confusion matrix provided in Table 1 indicates that there was a good balance between high and low quality data in the testing subset: 58.7 % high quality and 41.3 % low quality.

In comparison, the previously proposed agreement SQI had a sensitivity and specificity of 59.7 (56.3−62.9) and 74.9 (71.4−78.2) % respectively on the same data. It classified 66.0 % of the segments correctly, and misclassified 10.4 % of segments as high quality. The confusion matrix is provided in the Supplementary Material. The discriminatory performance of the novel SQI was superior to that of the agreement SQI, as shown by the McNemar test rejecting the null hypothesis of marginal homogeneity between the two SQIs (*p* < 0.001). Both the sensitivity and specificity of the novel SQI were significantly higher than that of the agreement SQI. Additional results for the novel and agreement SQIs across the different clinical settings in the *RRest-vent* testing subset are provided in the Supplementary Material.

For reference, during training on the *RRest-vent* training subset, the novel SQI had a sensitivity and specificity (95 % CIs) of 74.6 (71.9−77.2) and 87.6 (85.3−89.7) %, and the agreement SQI had a sensitivity and specificity (95 % CIs) of 48.0 (45.2−51.2) and 81.7 (78.7−84.0) %. These results are in keeping with the preliminary results reported in [23,26].

### 3.2 A comparison of RRs obtained using a RR algorithm and from a clinical monitor

Table 2 shows the performance of two methods for obtaining RRs from ImP segments deemed to be of high quality by the novel SQI: the *Count-Orig* RR algorithm, and obtaining RR estimates from the clinical monitor RRs. RR estimates were more precise when obtained using the RR algorithm, with limits of agreement of 0.0 ± 1.0 bpm and 0.1 ± 1.8 bpm on the two datasets. In comparison, when using clinical monitor RRs the limits of agreement were significantly wider (0.3 ± 3.7 bpm and -0.1 ± 6.0 bpm), indicating less precision. The results indicate that performance was significantly improved when using the RR algorithm, rather than using RRs provided by the clinical monitor. Indeed, the frequencies of erroneous RRs (those with an error of > 2.0 bpm, indicated by CP_2_) were only 1.4 % and 7.7 % when using the RR algorithm, compared to 15.1 % and 29.8 % when using RRs from the monitor. Furthermore, the frequencies of highly erroneous RRs which could affect treatment decisions (those with an error of > 5.0 bpm), were 0.1 % and 0.2 % when using the RR algorithm, compared to 3.1 % and 10.2 % when using the clinical monitor RRs. The reference and estimated RRs obtained using each method are shown in Fig. 3.

The impact of false positives on RR estimates was assessed by calculating RRs (using the *Count-orig* algorithm) from those segments falsely identified as high quality by the novel SQI in the *RRest-vent* testing subset. RR estimates derived from the 94 segments which were falsely identified as high quality had limits of agreement of 0.2 ± 2.1 bpm (within which 95 % of errors are expected to lie). In comparison, RR estimates derived from the 580 correctly identified high-quality segments had limits of agreement of -0.1 ± 0.7 bpm. The limits of agreement were significantly wider for RR estimates derived from low-quality segments, indicating less precision in those segments which were actually of low quality.

### 3.3 The frequency at which RRs were reported when using the novel SQI

The impact of the novel SQI on the proportion of segments for which RRs were reported was assessed using the real-world *RRest-mimic* dataset. Overall, the novel SQI identified 34.9 % of the 10,782 non-flat-line segments as high quality in this dataset (the 517 flat-line segments were excluded from the analysis). On a per subject basis, the novel SQI identified a median (lower – upper quartiles) of 32.7 (12.3–55.3) % of each subject’s non-flat-line segments as high quality. The RR algorithm estimated RRs from all of the segments deemed to be high quality, indicating that the novel SQI allowed RRs to be obtained for approximately one third of the time in this real-world setting. There was a median (lower – upper quartiles) of 64 (32–224) s between RRs obtained from high quality segments identified by the novel SQI. The most recent RR was less than five and ten minutes ago for 79.7 % and 89.6 % of the time respectively.

Fig. 4 shows four case studies demonstrating potential benefits of using the novel SQI and RR algorithm, explained in the figure caption.

## 4 Discussion

This study presented a novel SQI for use with the ImP signal. The SQI classifies periods of ImP signal as either high or low quality by identifying candidate breaths, assessing the physiological plausibility of the resulting breath-to-breath timings, and assessing the consistency of the signal morphology of each breath. The SQI was assessed on two datasets, across a range of clinical settings. It showed good performance for discriminating between high and low quality data, outperforming a previous technique from the literature. In addition, when using a RR algorithm, RRs derived from the segments identified as high quality by the novel SQI were highly precise and accurate across a wide range of RRs. A real-world assessment indicated that RRs could be obtained for approximately one third of the time when using the novel SQI. The results indicate that the SQI may confer benefit in high-dependency settings. In the future it may also be found to provide benefit when used with wearable sensors, in both hospital and community settings.

The criteria used by the SQI to discriminate between high- and low-quality data provide insight into the reasons for its performance. The SQI only deemed segments to be of high quality if: they did not exhibit high variation in breath durations, the majority of the segment was occupied by breaths with well defined peaks and troughs, and these breaths exhibited similar morphology. This indicates the key strength of the SQI: it identifies high quality segments in which there is not high variability in the breathing pattern over short periods (seconds), and in which RR can be accurately estimated. It seems likely this would have utility for detecting changes in RR which accompany acute deteriorations in monitored hospital patients, where current nurse observations are separated by several minutes or hours. However, it is not suitable for use in settings where either continuous RRs are required (such as for detection of apnea and respiratory arrests), or the breathing pattern is expected to be highly irregular (such as during ataxic breathing, cluster breathing, and potentially Cheyne-Stokes respiration [36], or in neonates).

This study builds on previous work on assessing the quality of physiological signals. The novel SQI was designed by adapting the approach presented by Orphanidou et al. for electrocardiogram (ECG) and photoplethysmogram (PPG) signals [11]. This approach was adapted for use with respiratory signals, and provided results comparable with previous work. The ability of the SQI to distinguish between high and low quality data (sensitivity and specificity of 78 and 82 % respectively on the *RRest-vent* testing subset) was not as high as when the approach was used with the ECG (94 and 97 %) and the PPG (91 and 95 %). However, the RRs estimated from segments deemed to be high quality had minimal bias and a precision of 1.0 and 1.8 bpm (2SD on each dataset), which is comparable to previous studies of gold-standard RR measurements *(e.g*. 1.3 bpm when using an oral-nasal pressure sensor in [24]), and a better performance than typically achieved when estimating RR from ECG or PPG signals [24]. Furthermore, the MAEs of 0.21 and 0.40 bpm observed when using the novel SQI with a RR algorithm in this study improves on the best MAE of 0.42 bpm reported in a comparison of previous algorithms without an SQI (in laboratory rather than clinical conditions) [25]. The high performance of estimated RRs can be attributed to the selection of high-quality segments, and the use of the *Count-orig* RR algorithm, which has previously been shown to provide superior performance to other RR estimation techniques [24,25,32]. Nonetheless, the observation that RRs were less precise in segments falsely identified as high quality by the novel SQI indicates that the SQI could be improved in the future.

The novel SQI may impact clinical practice in several settings. Its performance on data acquired from static bedside monitors in this study indicates that it may be suitable for a prospective clinical study in high-dependency settings. However, it is likely to confer greater benefit outside of the critical care setting, where there is a lower ratio of staff to patients. In areas such as the ambulatory ward, or home setting, the SQI could potentially improve the alert rate resulting from ImP monitoring using wearable sensors. The case studies demonstrated how it could reduce the false alert rate, and increase the true alert rate, which would reduce healthcare costs and improve patient safety respectively. However, a key limitation of this study is that the performance of the SQI has not been assessed on data from ambulatory patients. Therefore, further work is required to assess its performance in this setting before it could reasonably be used with wearable sensor data. Such studies could be performed using both ImP monitoring and reference respiratory monitoring (such as by a facemask) during rest and exercise: the Vortal dataset would be suitable for such studies [24,37]. In addition, the performance of the SQI was assessed against signal quality and breath annotations provided by a single annotator. Future studies could provide further, complementary evidence on its performance in additional clinical settings.

The SQI is also expected to have impact in the research setting. A recent review of techniques to estimate RR from the ECG and PPG identified the need to obtain reliable RRs from reference respiratory signals to evaluate the performance of ECG- and PPG-based RR algorithms [27]. Several datasets which have been previously used to develop RR algorithms contain reference ImP signals, such as the MGH/MF and MIMIC datasets [29,30,38]. The MIMIC dataset, containing data from critical care patients, is widely used for other purposes too [39]. The SQI is suitable for extracting reliable RRs from this dataset, increasing the scope of studies which can be conducted on the dataset.

## 5 Conclusions

The novel ImP SQI presented in this study was found to discriminate well between low- and high-quality data, and result in highly accurate and precise RR estimates when coupled with a high performance RR algorithm. The SQI was assessed in the critical care setting, and may confer clinical benefit for identifying acute deteriorations in that setting. It is also a valuable resource for future research, enhancing the value of existing datasets containing ImP signals. A Matlab ® implementation of the SQI is publicly available (see Supplementary Material). Importantly, the SQI is not suitable for use in settings where RRs are required continuously. Furthermore, it has not yet been assessed outside of the critical care setting, and in the presence of irregular breathing patterns. It could potentially have great benefit if used with wearable sensors, making this a promising avenue for future research.

## Supplementary Material

Supplementary material related to this article can be found, in the online version, at doi: https://doi.org/10.1016/j.bspc.2020.102339.

Data profile

Supplementary material

## Figures and Tables

**Table 1:** The discriminatory performance of the novel SQI, assessed on the RRest-vent testing subset. The confusion matrix for the novel SQI is shown, indicating the number of ImP signal segments in each category, and the percentage of segments deemed to be of high and low quality by manual annotations (bottom row).

		Actual Class (determined by manual annotation)	
		High Quality	Low Quality
Predicted Class (determined by novel SQI)	High Quality	615	99
	Low Quality	177	459
		58.7 %	41.3 %

**Table 2:** The performance of RRs estimated from segments deemed to be of high quality by the novel SQI. Results are reported for each dataset, when: (i) using the Count-Orig RR algorithm to estimate RRs; and (ii) obtaining RR estimates from the clinical monitor RRs. CI: confidence interval. Statistics are as defined in Section 2.5.

	*RRest-vent* testing subset		*RRest-mimic dataset*	
	Novel SQI & RR Algorithm	Novel SQI & Clinical Monitor RRs	Novel SQI & RR Algorithm	Novel SQI & Clinical Monitor RRs
Bias [bpm] (95 % CI)	0.0 (-0.2 – 0.1)	0.3 (-0.2 – 0.7)	0.1 (-0.1 – 0.2)	− 0.1 (-0.8 – 0.5)
2SD [bpm] (95 % CI)	1.0 (0.8–1.2)	3.7 (2.9–4.4)	1.8 (1.5–2.1)	6.0 (4.9–7.1)
CP_2_ [%]	98.6	84.9	92.3	70.2
iCP_5_ [%]	0.1	3.1	0.2	10.2
MAE [bpm]	0.21	1.04	0.40	1.90
Number of windows	714	709	452	423

## Abbreviations

bpm: breaths per minute
CI: confidence interval
ImP: impedance pneumography
ICU: intensive care unit
MAE: mean absolute error
R: correlation coefficient
RR: respiratory rate
SQI: signal quality index
