# Supplementary material for: An impedance pneumography signal quality index: Design, assessment and application to respiratory rate monitoring
Source: Biomed Signal Process Control. Author manuscript; Available in PMC 2021 Sep 1. (PMC7611038; doi:10.1016/j.bspc.2020.102339)
Supplement: Supplementary material [file EMS126271-supplement-Supplementary_material.docx]

**Supplementary Material**

# Additional Results

The confusion matrix from which the discriminatory performance of the novel SQI was assessed was presented in Table 1 of the main text. The corresponding confusion matrix for the agreement SQI is present in Table S3.

Table S3: The discriminatory performance of the agreement SQI, assessed on the RRest-vent testing subset. The confusion matrix for the agreement SQI is shown, indicating the number of ImP signal segments in each category, and the percentage of segments deemed to be of high and low quality by manual annotations (bottom row).

|  | | Actual Class  (determined by manual annotation) | |
| --- | --- | --- | --- |
|  |  | High Quality | Low Quality |
| Predicted Class  (determined by novel SQI) | High Quality | 473 | 140 |
|  | Low Quality | 319 | 418 |
|  |  | 58.7% | 41.3% |

The discriminatory performance of the novel SQI was summarised for the entire *RRest-vent* dataset in Table 1 and in the main text. The corresponding results separated according to clinical setting, for both the novel SQI and the agreement SQI, are provided in Table S4 and Table S5 respectively. The performance of RR estimates obtained from segments deemed to be of high quality by each SQI is also provided for completeness. For comparison, the performance of RR estimates obtained from segments manually deemed to be of high quality is shown in Table S6.

Table S4: The performance of the novel signal quality index (SQI) on the RRest-vent testing subset.

|  | All | Ventilated in ICU | Unassisted in ICU | Shortly before ambulatory ward |
| --- | --- | --- | --- | --- |
| *Assessing signal quality* | | | | |
| Sensitivity [%] (95% CI) | 77.7  (74.9 – 80.4) | 82.4  (77.6 – 86.8) | 76.7  (70.0 – 81.8) | 74.0  (68.9 – 78.7) |
| Specificity [%] (95% CI) | 82.3  (79.0 – 85.2) | 74.0  (66.8 – 80.3) | 90.7  (86.3 – 94.3) | 79.2  (72.4 – 85.4) |
| Number of segments | 1,350 | 450 | 450 | 450 |
| *The performance of RR estimates obtained using the Count-Orig RR algorithm* | | | | |
| Bias [bpm] (95% CI) | 0.0  (-0.2 – 0.1) | -0.1  (-0.3 - 0.1) | 0.0  (-0.3 - 0.3) | 0.0  (-0.2 – 0.2) |
| 2SD [bpm] (95% CI) | 1.0  (0.8 – 1.2) | 0.9  (0.6 – 1.2) | 1.4  (0.9 – 1.9) | 0.8  (0.5 – 1.1) |
| CP_2_ [%] | 98.6 | 99.3 | 97.4 | 98.8 |
| iCP_5_ [%] | 0.1 | 0.0 | 0.5 | 0.0 |
| MAE [bpm] | 0.21 | 0.23 | 0.24 | 0.18 |
| Number of segments | 714 | 271 | 192 | 251 |
| *The performance of RR estimates obtained from clinical monitor RRs* | | | | |
| Bias [bpm] (95% CI) | 0.3  (-0.2 – 0.7) | 0.3  (-0.5 – 1.1) | 0.3  (-0.7 – 1.3) | 0.2  (-0.4 – 0.8) |
| 2SD [bpm] (95% CI) | 3.7  (2.9 – 4.4) | 3.6  (2.3 – 5.0) | 4.5  (2.8 – 6.3) | 2.9  (1.9 – 4.0) |
| CP_2_ [%] | 84.9 | 84.5 | 81.2 | 88.3 |
| iCP_5_ [%] | 3.1 | 3.3 | 5.2 | 1.2 |
| MAE [bpm] | 1.04 | 0.92 | 1.36 | 0.94 |
| Number of windows | 709 | 271 | 191 | 247 |

Table S5: The performance of the agreement signal quality index (SQI) on the RRest-vent testing subset.

|  | All | Ventilated in ICU | Unassisted in ICU | Shortly before ambulatory ward |
| --- | --- | --- | --- | --- |
| *Assessing signal quality* | | | | |
| Sensitivity [%] (95% CI) | 59.7  (56.3 – 62.9) | 48.7  (42.7 – 54.4) | 63.2  (56.9 – 69.1) | 67.2  (62.0 – 72.2) |
| Specificity [%] (95% CI) | 74.9  (71.4 – 78.2) | 71.8  (64.2 – 77.7) | 81.5  (76.3 – 86.3) | 68.8  (61.5 – 75.3) |
| Number of segments | 1,350 | 450 | 450 | 450 |
| *The performance of RR estimates obtained using the Count-Orig RR algorithm* | | | | |
| Bias [bpm] (95% CI) | 0.4  (0.0 – 0.8) | 0.4  (-0.3 – 1.1) | 0.3  (-0.2 – 0.8) | 0.5  (-0.3 – 1.2) |
| 2SD [bpm] (95% CI) | 3.0  (2.4 – 3.7) | 2.9  (1.8 – 4.1) | 2.3  (1.4 – 3.2) | 3.5  (2.2 – 4.8) |
| CP_2_ [%] | 89.6 | 87.4 | 89.1 | 91.5 |
| iCP_5_ [%] | 1.6 | 1.6 | 0.5 | 2.4 |
| MAE [bpm] | 0.60 | 0.62 | 0.53 | 0.64 |
| Number of segments | 613 | 183 | 183 | 247 |
| *The performance of RR estimates obtained from clinical monitor RRs* | | | | |
| Bias [bpm] (95% CI) | 0.4  (-0.1 – 1.0) | 0.4  (-0.6 – 1.3) | 0.4  (-0.9 – 1.6) | 0.5  (-0.3 – 1.3) |
| 2SD [bpm] (95% CI) | 4.5  (3.6 – 5.5) | 4.1  (2.5 – 5.8) | 5.7  (3.6 – 7.9) | 3.8  (2.4 – 5.2) |
| CP_2_ [%] | 80.0 | 80.9 | 74.6 | 83.3 |
| iCP_5_ [%] | 4.9 | 3.8 | 7.2 | 4.1 |
| MAE [bpm] | 1.32 | 1.13 | 1.72 | 1.18 |
| Number of segments | 610 | 183 | 181 | 246 |

Table S6: The performance of RR estimates obtained from segments manually identified as high quality in the RRest-vent testing subset.

|  | All | Ventilated in ICU | Unassisted in ICU | Shortly before ambulatory ward |
| --- | --- | --- | --- | --- |
| *The performance of RR estimates obtained using the Count-Orig RR algorithm* | | | | |
| Bias [bpm] (95% CI) | 0.0  (-0.2 – 0.1) | -0.1  (-0.3 - 0.1) | 0.0  (-0.2 - 0.3) | 0.0  (-0.2 – 0.3) |
| 2SD [bpm] (95% CI) | 1.1  (0.9 – 1.3) | 1.0  (0.6 – 1.3) | 1.3  (0.8 – 1.8) | 1.1  (0.7 – 1.5) |
| CP_2_ [%] | 97.9 | 98.7 | 96.9 | 98.0 |
| iCP_5_ [%] | 0.0 | 0.0 | 0.0 | 0.0 |
| MAE [bpm] | 0.24 | 0.25 | 0.25 | 0.22 |
| Number of segments | 681 | 238 | 191 | 252 |
| *The performance of RR estimates obtained from clinical monitor RRs* | | | | |
| Bias [bpm] (95% CI) | 0.2  (-0.3 – 0.7) | 0.1  (-0.7 – 0.9) | 0.3  (-0.9 – 1.4) | 0.2  (-0.5 – 0.8) |
| 2SD [bpm] (95% CI) | 3.9  (3.1 – 4.7) | 3.7  (2.4 – 5.1) | 5.1  (3.1 – 7.1) | 3.0  (1.9 – 4.2) |
| CP_2_ [%] | 84.9 | 86.1 | 79.5 | 88.0 |
| iCP_5_ [%] | 3.1 | 2.5 | 5.8 | 1.6 |
| MAE [bpm] | 1.07 | 0.88 | 1.47 | 0.96 |
| Number of segments | 677 | 238 | 190 | 249 |

# Reproduction Instructions

The curation and analyses of the *RRest-mimic* subset reported in this paper can be reproduced as follows.

***Reproducing the analysis***
These steps can be used to quickly reproduce the analysis using the curated and annotated dataset.

1. Download the curated and annotated dataset from [Zenodo](https://doi.org/10.5281/zenodo.3973770) using this [direct download link](https://zenodo.org/record/3973771/files/mimic_imp_sqi_data.mat?download=1).
2. Run the analysis using the [*run_imp_sqi_mimic.m*](https://zenodo.org/record/3973771/files/run_imp_sqi_mimic.m?download=1) script.

***Reproducing data extraction, curation and analysis***
These steps include downloading the raw data files, extracting data from these files, collating the dataset, manually annotating the data, and performing the analysis.

1. Use the [*ImP_SQI_mimic_data_importer.m*](https://zenodo.org/record/3973771/files/ImP_SQI_mimic_data_importer.m?download=1) script to download raw MIMIC data files from PhysioNet, and collate them into a single Matlab ® file.
2. Prepare the dataset for manual annotation by running the [*run_imp_sqi_mimic.m*](https://zenodo.org/record/3973771/files/run_imp_sqi_mimic.m?download=1) script.
3. Manually annotate the signals by running the [*run_mimic_imp_annotation.m*](https://zenodo.org/record/3973771/files/run_imp_sqi_mimic.m?download=1) script - the annotations are stored in separate files (the original annotation files are available [here](https://zenodo.org/record/3974113/files/2019_annotations.zip?download=1)).
4. Import the manual annotations into the collated data file by re-running the [*ImP_SQI_mimic_data_importer.m*](https://zenodo.org/record/3973771/files/ImP_SQI_mimic_data_importer.m?download=1) script.
5. Run [*run_imp_sqi_mimic.m*](https://zenodo.org/record/3973771/files/run_imp_sqi_mimic.m?download=1) to perform the analysis described in the publication.
